# A preliminary report of longitudinal white matter alterations in patients with end-stage renal disease: A three-year diffusion tensor imaging study

**DOI:** 10.1371/journal.pone.0215942

**Published:** 2019-04-30

**Authors:** Ming-Chung Chou, Chih-Hung Ko, Tsyh-Jyi Hsieh, Jer-Ming Chang, Wei-Shiuan Chung

**Affiliations:** 1 Department of Medical Imaging and Radiological Sciences, Kaohsiung Medical University, Kaohsiung, Taiwan; 2 Department of Healthcare Administration and Medical Informatics, Kaohsiung Medical University, Kaohsiung, Taiwan; 3 Department of Medical Research, Kaohsiung Medical University Hospital, Kaohsiung, Taiwan; 4 Department of Psychiatry, College of Medicine, Kaohsiung Medical University, Kaohsiung, Taiwan; 5 Department of Psychiatry, Kaohsiung Medical University Hospital, Kaohsiung Medical University, Kaohsiung, Taiwan; 6 Department of Psychiatry, Kaohsiung Municipal Siaogang Hospital, Kaohsiung Medical University, Kaohsiung, Taiwan; 7 Department of Medical imaging, Chi Mei Medical Center, Tainan, Taiwan; 8 Department of Radiology, College of Medicine, Kaohsiung Medical University, Kaohsiung, Taiwan; 9 Department of Renal Care, College of Medicine, Kaohsiung Medical University, Kaohsiung, Taiwan; 10 Department of Internal Medicine, Kaohsiung Medical University Hospital, Kaohsiung Medical University, Kaohsiung, Taiwan; 11 Department of Medical Imaging, Kaohsiung Municipal Siaogang Hospital, Kaohsiung Medical University, Kaohsiung, Taiwan; 12 Department of Medical Imaging, Kaohsiung Medical University Hospital, Kaohsiung Medical University, Kaohsiung, Taiwan; University of California, San Francisco, UNITED STATES

## Abstract

**Purpose:**

End-stage renal disease (ESRD) patients exhibit silent white-matter alterations after long-term hemodialysis, which may be due to ESRD itself or the hemodialysis. The purpose of this study was to investigate the longitudinal white-matter alterations in the ESRD patients under 3-year long-term hemodialysis using voxel-wise analysis of diffusion tensor imaging (DTI).

**Materials and methods:**

15 ESRD patients and 15 age-matched healthy controls participated in this study. Due to the development of abnormal brain lesions in some cases, 13 ESRD patients and 13 age-matched healthy controls were enrolled and underwent cognitive function assessment and DTI acquisition at two-time points separated by 3 years. Voxel-based analysis was performed to globally detect white-matter alterations between the two groups as well as between the two scans within the two groups.

**Results:**

In the ESRD patients, diffusivity indices were significantly increased and the fractional anisotropy was significantly decreased in both scans, as compared with healthy controls. Longitudinal comparisons showed significant white-matter alterations in healthy controls in three years, but little or no significant alterations were noted in the ESRD patients after additional 3-year hemodialysis.

**Conclusion:**

Poorer white matter integrity and cognitive function are noted in ESRD patients and the toxic effect of ESRD may be the major factor of white matter alterations.

## Introduction

End-stage renal disease (ESRD) is defined as the final stage of chronic kidney disease, with a permanent loss of >90% of normal renal function [1]. In the ESRD patients, urea and toxic metabolites are accumulated in the blood and tissues, which in turn leads to multiple organ dysfunction. In recent years, hemodialysis has been the most common treatment to remove excess metabolic waste from the body. However, the ESRD patients on dialysis may still develop uremic neuropathies such as osmotic demyelination syndrome, dialysis disequilibrium syndrome, and cerebrovascular diseases [2–4]. Thus, it is important to monitor changes in the brain tissue in such patients.

Previous studies used magnetic resonance imaging (MRI) to detect abnormalities in the brain tissue caused by hemodialysis. These studies showed T2 hyperintensities in both the supra- and infra-tentorial regions in the ESRD patients immediately after hemodialysis, whereas long-term hemodialysis gradually normalizes the T2 signals in these altered regions [5, 6]. Another study performed diffusion-weighted imaging (DWI) to measure subtle changes in the apparent diffusion coefficient (ADC) in the brain tissue of the ESRD patients immediately after hemodialysis [7]. They showed that hemodialysis immediately and significantly increased ADC in some regions of the brain. Because the effects of transient hemodialysis may be different from those of long-term hemodialysis, additional studies performed diffusion tensor imaging (DTI) to detect white-matter alterations in the ESRD patients on hemodialysis for more than 1 year [8, 9]. They showed that DTI-derived fractional anisotropy (FA) was significantly decreased and axial diffusivity (AD), radial diffusivity (RD), and mean diffusivity (MD) were significantly increased in white-matter tissue after long-term hemodialysis.

Although significant correlations were noted between the DTI indices and duration of hemodialysis in the previous cross-sectional studies [8, 9], it remains unclear whether the major cause of white matter alterations in the ESRD patients is ESRD itself or the hemodialysis, which might be better understood from a longitudinal study. In addition, because previous reports showed that long-term hemodialysis helped normalize the T2 signals in the altered brain regions [5, 6], the white matter alterations might be slow down in the ESRD patients after long-term hemodialysis. Therefore, the purpose of this study was to monitor the longitudinal changes of global white-matter alterations in the ESRD patients on regular hemodialysis and to understand whether 3-year regular hemodialysis could lead to additional white matter alterations in the ESRD patients.

## Materials and methods

### Subjects

**Fig 1:**
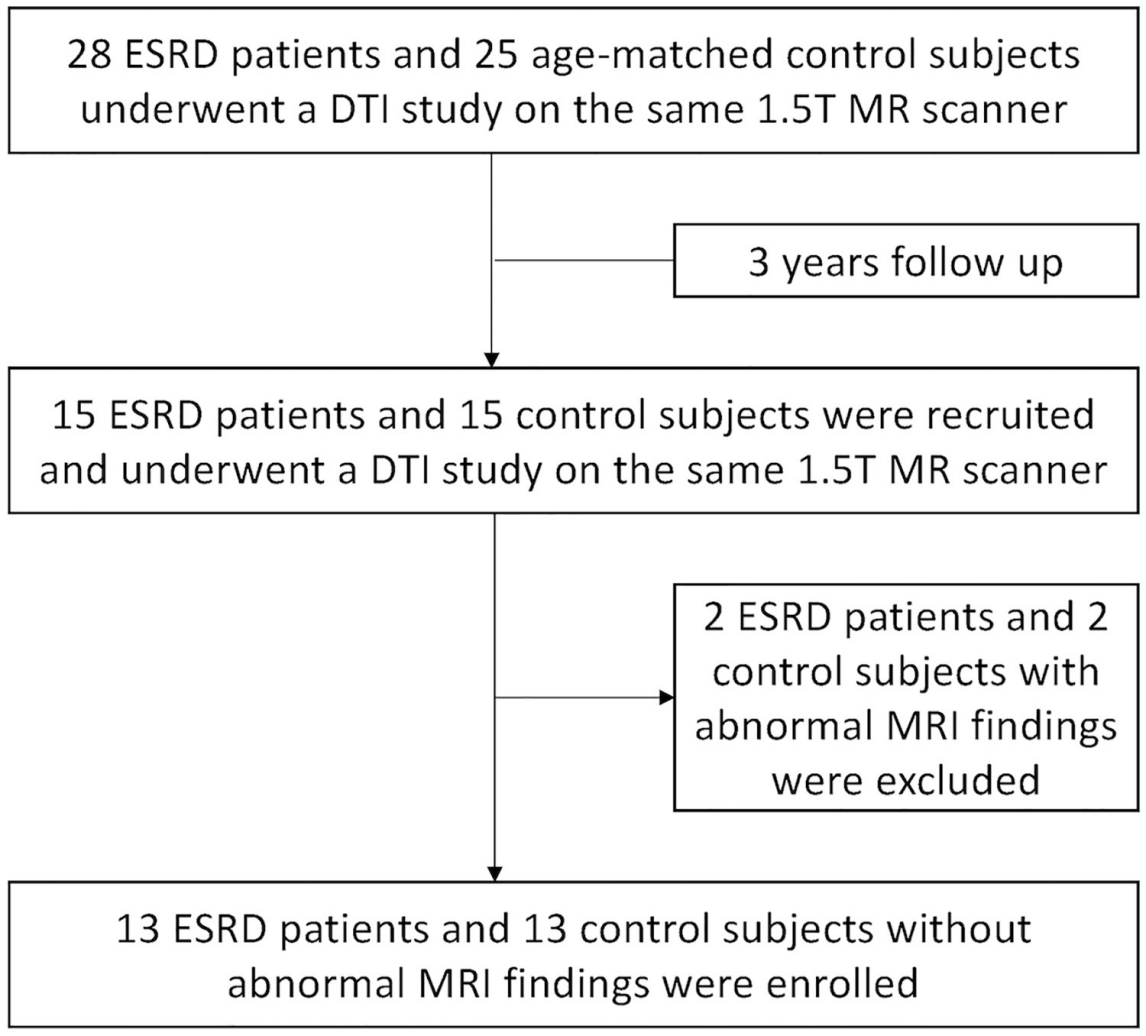
Flowchart of follow-up. The flowchart shows the number of volunteers participating in the inceptive examination and the 3-year follow-up, where some participants failed to undergo follow-up assessments. The 15 ESRD patients and 15 age-matched control subjects who participated from the inception through the 3-year follow-up constituted the study group. Finally, 13 ESRD patients and 13 age-matched control subjects were enrolled after excluding 2 ESRD patients and 2 healthy control subjects due to the development of abnormal brain lesions during the 3 years.

This prospective study was approved by the Kaohsiung Medical University Chung-Ho Memorial Hospital Institutional Review Board (KMUH-IRB-980232) and written informed consent was obtained from each participant. 15 ESRD patients and 15 age-matched healthy control subjects were recruited. To avoid possible confounding factors, the participants with interval development of diabetes, alcoholism, drug abuse, psychiatric disorders, or major neurologic disorders (severe head injury, stroke, epilepsy, or visible lesions) were excluded. In this study, 2 ESRD patients and 2 healthy control subjects were excluded due to the development of abnormal brain lesions. Finally, 13 ESRD patients and 13 age-matched control subjects were enrolled with planned reassessment after 3 years, as shown in Fig 1. For the ESRD patients, the last regular hemodialysis was conducted 2 days before the MRI examination. Table 1 lists the demographic characteristics of the enrolled participants. After obtaining informed consent, we used the cognitive abilities screening instrument (CASI) assessment and performed an approximately 30-min-long overall evaluation of cognitive function in all participants [10]. This assessment covers nine domains of cognition, including attention, concentration, orientation, short-term memory, long-term memory, language, visual construction, list-generating fluency, and abstract thinking judgment, and is commonly used in the clinical evaluation of cognitive changes.

**Table 1:** Demographic characteristics of participants.

	ESRD patients		Control subjects	
	First	Second	First	Second
Gender (M/F)	7 / 6		4 / 9	
Age (y/o)	36.9 ± 8.1	39.9 ± 8.1	40.3 ± 6.3	43.3 ± 6.3
Dialysis Duration (y)	7.2 ± 3.4	10.2 ± 3.4	N/A	
CASI score	91.2 ± 7.8*	89.8 ± 5.8†	97 ± 3.5*	95.2 ± 2.5†

Demographic characteristics of ESRD patients and control subjects at two MRI scans separated by 3 years.

* indicate statistically significant difference (*P* < 0.05) between the ESRD and controls groups in the first scan.

^†^ indicate statistically significant difference (*P* < 0.05) between the ESRD and controls groups in the second scan.

### Data acquisition

All brain MRI data were acquired using a 1.5T MR scanner (Signa Excite, GE Medical Systems). After tri-planar scans and the acquisition of calibration data for ASSET parallel imaging, 20 axial T1WI, T2WI, and T2-FLAIR images were sequentially obtained from each participant. Because previous MRI studies showed that patients who had dialysis disequilibrium syndrome or osmotic dysmyelination after undergoing hemodialysis may exhibit T2 hyper signal intensities [5, 6, 11], we used anatomical images to diagnose pre-existing lesions in patients, and those who were diagnosed with lesions were excluded from this study.

For those patients without pre-existing lesions, DTI acquisitions were performed using a single-shot, twice-refocused, spin-echo, echo-planar, diffusion-weighted sequence with an 8-channel phased array neurovascular coil. Thirty contiguous axial slices covering the whole brain were acquired, with the slice orientation parallel to the line joining the anterior and posterior commissures. Other imaging parameters were as follows: TR/TE = 8000/82.8 ms, matrix size = 128 × 128, b = 1000 s/mm^2^, the number of the non-collinear diffusion direction = 33, the number of b0 = 1, FOV = 240 × 240 mm, NEX = 1, ASSET factor = 2.0, and slice thickness = 4.4 mm. The scan time for the DTI acquisition was 4 min and 48 s.

### DTI analysis

All DTI data were transferred to a standalone workstation and processed using FSL (FMRIB Software Library, Oxford) to obtain diffusion tensor maps. First, the eddy current distortions were corrected using affine registration to minimize the diffusion gradient-induced eddy current distortions in 33 DWIs, with the b0 image as the reference. Subsequently, a diffusion tensor was fitted with the least square estimation on a voxel-by-voxel basis to obtain three eigenvalues, from which AD, RD, MD, and FA values were calculated for comparisons.

### Voxel-wise DTI analysis

**Fig 2:**
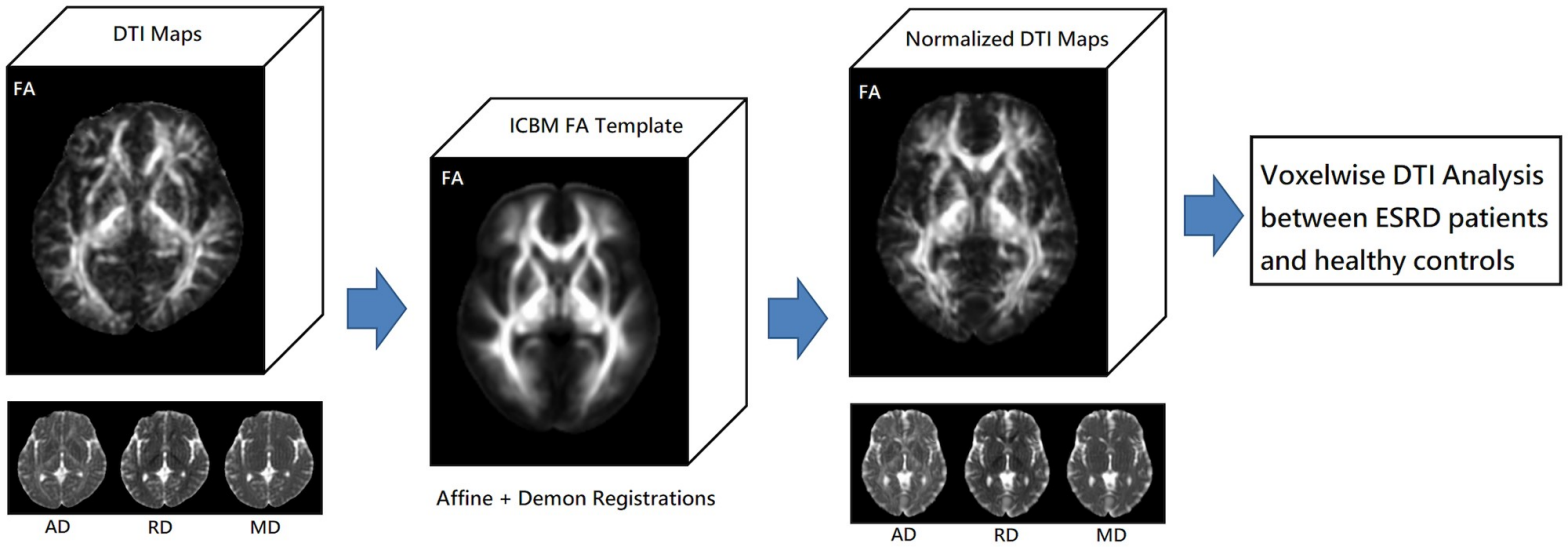
The post-processing steps of voxelwise DTI analysis. After scalp stripping, individual whole-brain DTI maps are spatially normalized to an international consortium for brain mapping (ICBM) FA template using linear affine and non-linear diffeomorphic demon registrations. Subsequently, the normalized DTI maps are utilized in voxelwise DTI analysis to show the differences between ESRD patients and healthy controls.

In the voxel-based analysis, the whole brain FA maps were spatially normalized to an international consortium for brain mapping FA template [12] using affine registration to minimize global differences between individual and template images. In addition, because local difference may still exist after affine registration and bias the statistical results, this study further utilized non-linear diffeomorphic demon registration [13,14], which was demonstrated to outperform several non-rigid registration methods including FNIRT (https://fsl.fmrib.ox.ac.uk/fsl/fslwiki/FNIRT) used in tract-based spatial statistics [15], to further minimize local differences between individual and template images. The displacement maps generated from the image registrations were then used to spatially normalize the corresponding AD, RD, and MD maps. The post-processing steps of voxelwise DTI analysis are shown in Fig 2.

### Statistical analysis

Subsequently, the voxel-wise comparison was performed using Statistical Parametric Mapping 8 (Wellcome Department of Cognitive Neurology, London, UK) on a MATLAB platform (MathWorks, Natick, Mass). The following five different statistical analyses were performed in this study: 1) a Mann-Whitney test and a Wilcoxon signed rank to show the difference in clinical data between the two groups and between the two scans, respectively, 2) a voxelwise two-sample *t*-test with age and gender as covariates to show the differences between the ESRD patients and the healthy subjects in the DTI indices, 3) a voxelwise flexible factorial design to show the interaction of group by time in the DTI indices in the ESRD patients and the healthy subjects, respectively, using a 2 x 2 design-matrix which consisted of two groups (controls and patients) and two conditions (time points: 0 and 3 years) with age and gender as covariates. In the significant regions, a Mann-Whitney U test was further performed to compare the DTI indices between the two groups. 4) a voxelwise multiple regression analysis to reveal the age-dependent white matter changes in the ESRD patients and the healthy subjects, respectively, and 5) Pearson’s correlational analysis to reveal the relationship between the DTI indices and CASI scores in regions with significance. In the voxelwise analysis, regions with statistical significance were displayed as red-yellow colors and superimposed on averaged maps of DTI indices of all healthy subjects for better visualization, and DTI results were reported if cluster-level corrected *P*<0.05 (*P* <0.01 and cluster size >100 voxels). The results of clinical data and the correlational analysis were reported if *P*-values were <0.05.

## Results

### Demographic characteristics of participants

In this study, sex and age were not significantly different between the two groups. Mann-Whitney test showed that the CASI scores of the ESRD patients were significantly lower than that of the healthy controls at the first and the follow-up scans. Although the CASI scores of both ESRD patients and control subjects in the follow-up scan were lower than that in the first scan, the Wilcoxon Signed-rank test did not show significant changes of CASI scores between the first and follow-up scans in both ESRD patients and control subjects (Table 1). Moreover, no significant correlations were observed between the CASI scores, age, sex, or dialysis duration in the ESRD patients and normal controls.

### Group comparisons of the first scan

**Fig 3:**
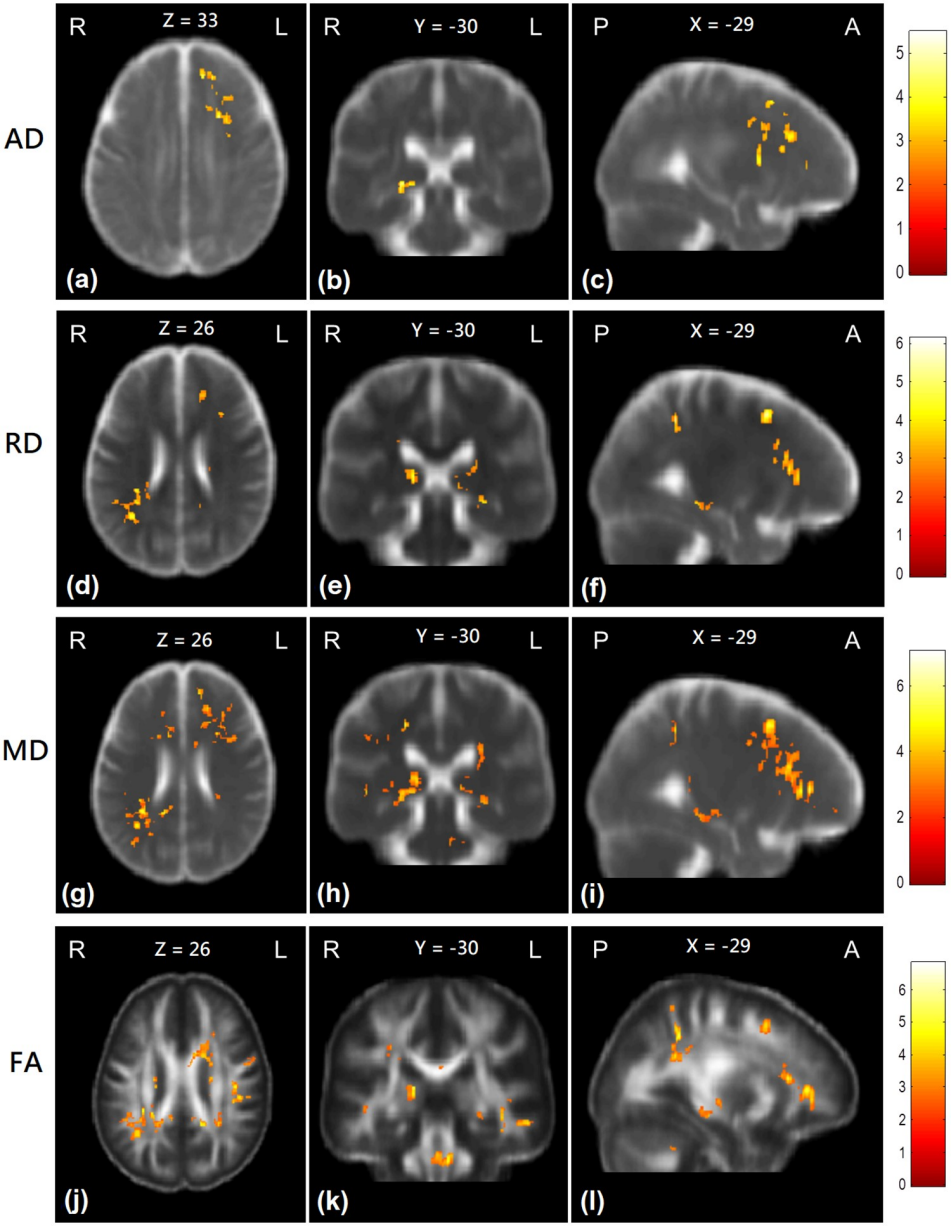
Group comparisons of the DTI indices between patient and control groups at the first scan. Red-yellow colors show the regions with significantly higher AD (a-c), RD (d-f), MD (g-i), and significantly lower FA (j-l) values than control subjects. Color bars in the right-hand side indicate T-value.

At the first scan, the ESRD patients had significantly higher AD values in the bilateral anterior frontal lobes and right posterior limb of internal capsule than healthy controls. RD values in bilateral anterior frontal and occipital lobes and the left posterior corona radiata were significantly higher in the ESRD patients than in healthy controls. MD values in the bilateral anterior frontal and parietal lobes were widespread significantly higher in the ESRD patients than in healthy controls. Moreover, FA values of the ESRD patients in the bilateral anterior frontal and parietal lobes, bilateral inferior longitudinal fasciculus, and splenium of corpus callosum were significantly lower than that of healthy controls (Fig 3 and S1 Table).

### Group comparisons of the second scan

**Fig 4:**
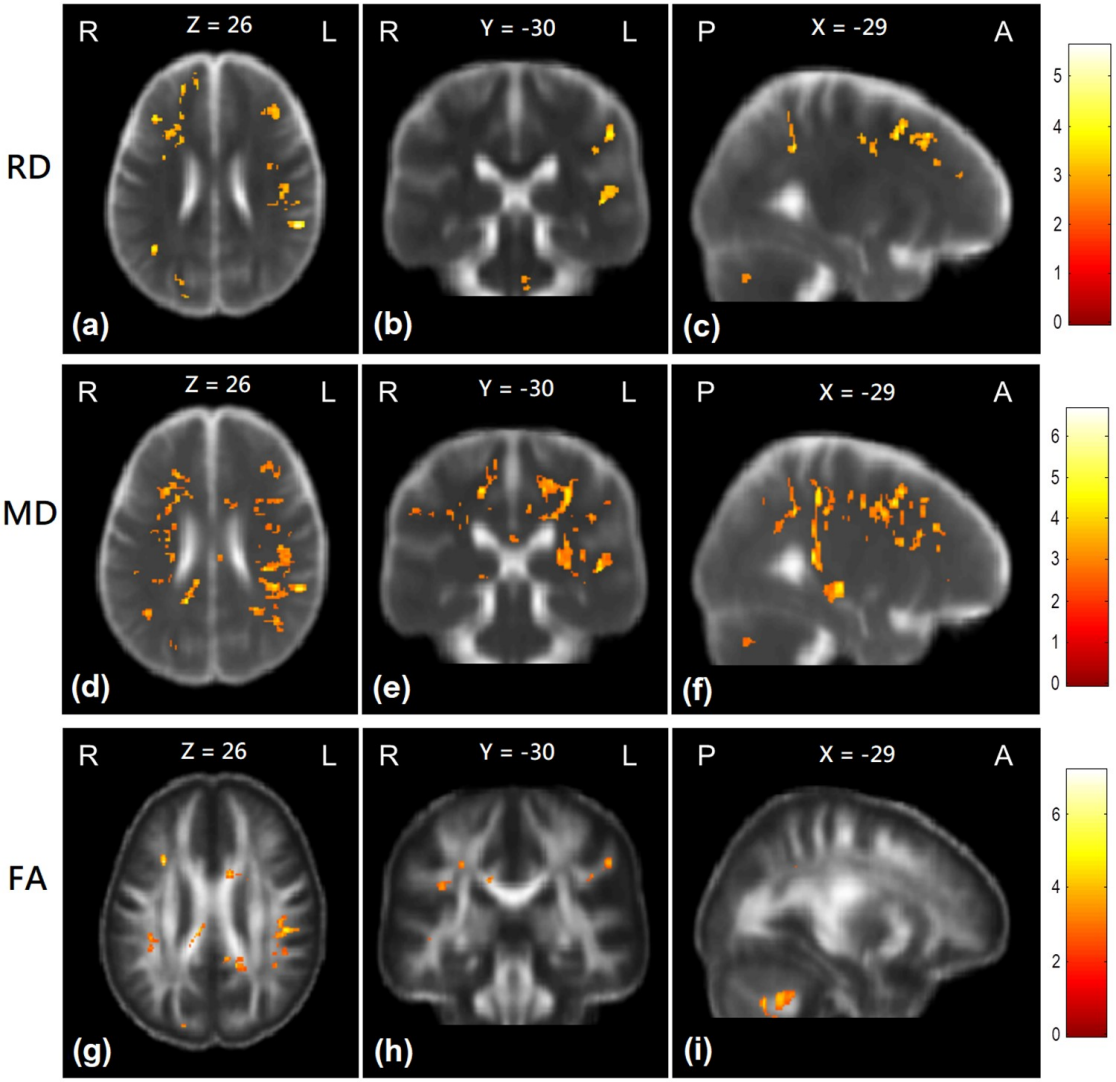
Group comparisons of the DTI indices between patient and control groups at the second scan. Red-yellow colors show the regions with significantly higher RD (a-c), MD (d-f), and significantly lower FA (g-i) values than control subjects. Color bars in the right-hand side indicate T-value.

At the second scan, the ESRD patients who underwent additional 3-year hemodialysis did not have significantly different AD values from healthy controls. However, RD values in bilateral superior frontal and parietal lobes were sporadically significantly higher in the ESRD patients than in healthy controls. MD values in the bilateral superior frontal and parietal lobes of the ESRD patients were significantly higher than that of healthy controls. Moreover, the results also showed that FA values of the ESRD patients were significantly lower than healthy controls in bilateral parietal lobes, right superior longitudinal fasciculus, and bilateral forceps major (Fig 4 and S2 Table).

### Interaction analysis of group by time

**Fig 5:**
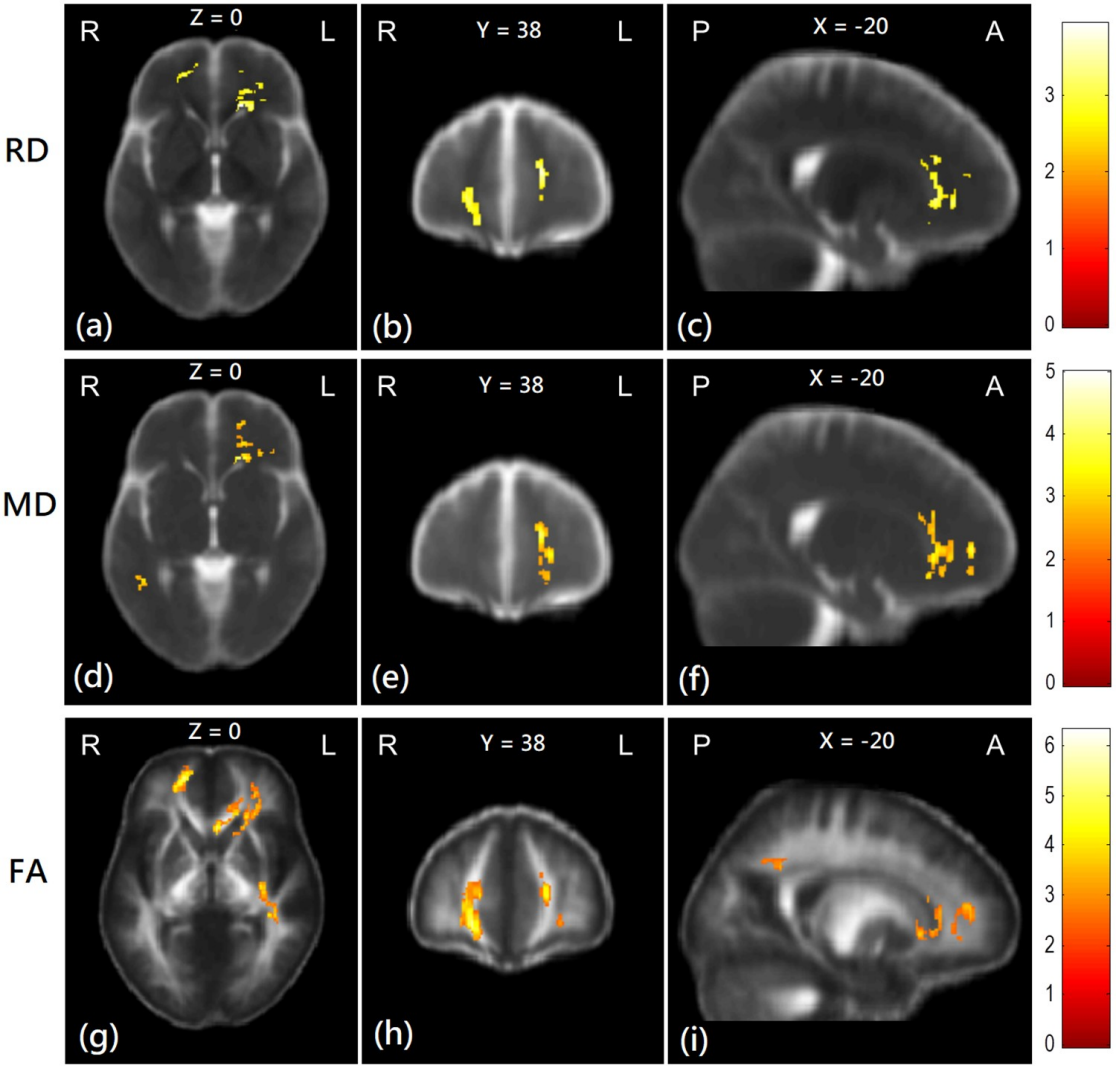
Interaction analysis of the DTI indices of healthy controls between the two scans. Red-yellow colors show the regions with significant increase of RD (a-c), MD (d-f), and FA (g-i) values in healthy controls after 3-years. Color bars in the right-hand side indicate T-value.

The interaction analysis (flexible factorial design) between the group (control and patient) and condition (time points: 0 and 3 years) showed that no significant change of AD values in both the ESRD patients and healthy controls between the two time points separated by 3 years. RD values of healthy controls in bilateral frontal lobes were significantly increased after 3 years, but no significant RD change was noted in the ESRD patients after additional 3-year hemodialysis. MD values of healthy controls were significantly increased in the left forceps minor, right posterior corona radiata, and right sagittal stratum, but were significantly decreased only in the left superior corona radiata after 3 years. Again, no significant difference was noted in the ESRD patients after additional 3-year hemodialysis. FA values of the healthy controls were significantly decreased in the bilateral forceps minor, right splenium of corpus callosum, left sagittal stratum and left forceps major. However, no significant FA change was noted in the ESRD patients after an additional 3 years of hemodialysis (Fig 5).

**Fig 6:**
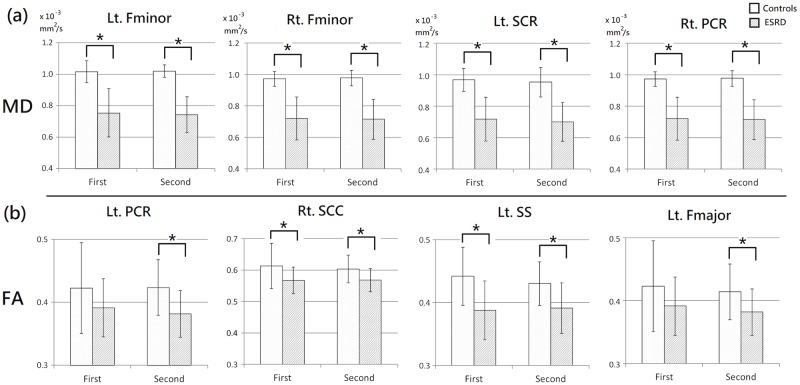
Comparisons of DTI indices between ESRD patients and healthy controls in significant regions of interaction analysis. In the significant regions, MD (a) and FA (b) values were significantly higher in healthy controls than in ESRD patients. Asterisks (*) indicate statistical significance (*P* < 0.05). Fmajor = forceps major; Fminor = forceps minor; SCC = splenium of corpus callosum; SCR = superior corona radiata; PCR = posterior corona radiata; SS = sagittal stratum; Lt. = left; Rt. = right.

Moreover, in the significant regions, the post-hoc comparisons showed significantly different MD and FA values in multiple white matter regions between the two groups in the two scans (Fig 6), which however were not significantly different in the voxelwise analysis.

### White matter demyelination

According to a previous study that demyelination could be characterized by increased RD with unchanged AD in white matter tissues [16], this study further detected white matter regions with increased RD and unchanged AD in the healthy controls over 3 years. The results showed that no significant worsening of white matter demyelination occurred in the ESRD patients in the period of three years, but there was significant longitudinal white matter demyelination detected in bilateral anterior frontal lobes, left posterior corona radiata, and left forceps major of healthy controls (Fig 5). Pearson’s correlational analysis further showed significant negative correlations between the RD and CASI scores in these demyelinated white matter regions of the healthy controls (Table 2).

**Table 2:** Correlation Coefficients between DTI indices and CASI scores.

Regions	Correlation coefficient		
	RD	MD	FA
Rt. PCR	-0.4424*	-0.3419	0.3251
Rt. Fmajor	-0.4142*	0.0146	0.3076
Lt. Fminor	-0.5040*	-0.3864	0.3085
Rt. Fminor	-0.3827*	-0.1874	0.1270

The table shows the correlation coefficients between DTI indices and CASI scores of healthy controls in demyelinated white-matter regions characterized as increased RD and unchanged AD.

* P < 0.05

PCR = posterior corona radiata; Fmajor = forceps major; Fminor = forceps minor

### Age-dependent white matter changes

**Fig 7:**
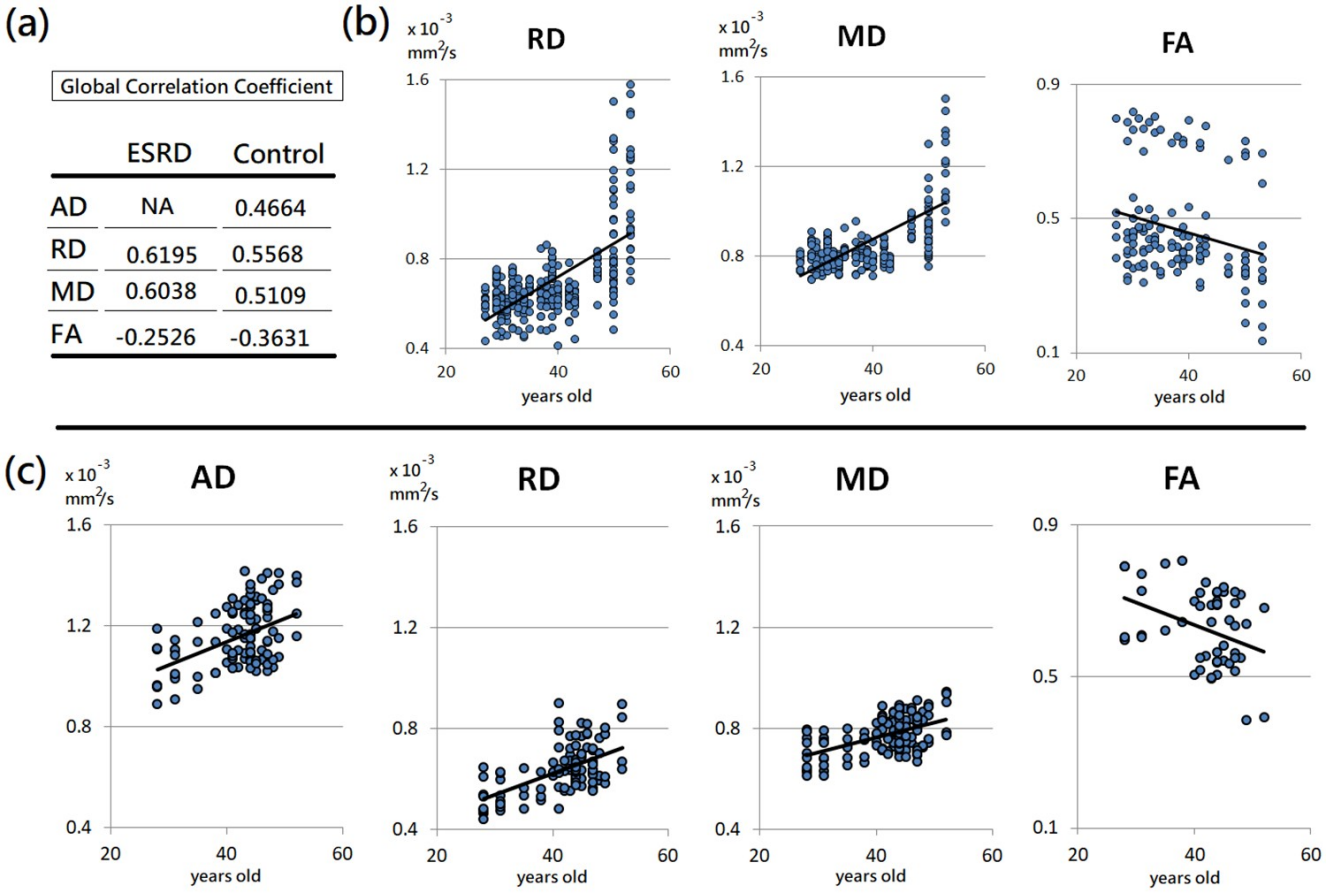
Correlations of DTI indices and age in ESRD patients and healthy controls. In whole-brain significant clusters, the correlation analysis showed significant correlations of DTI indices and age (a) in both ESRD patients (b) and healthy controls (c).

The multiple regression analysis revealed significant positive correlations between AD, RD, MD, and age, and a significant negative correlation between FA and age in multiple white matter regions in the healthy subjects (S1 Fig). The significant regions were mainly in the frontal lobe, including genu of corpus callosum, bilateral anterior corona radiata, and bilateral superior corona radiata. In the ESRD patients, the significant positive correlations were found between RD, MD, and age, and a significant negative correlation between FA and age in more widespread white matter regions (S2 Fig). The significant regions included bilateral anterior, superior, and posterior corona radiata, sagittal stratum, and forceps major. However, no significant correlation was noted between AD and age in the patients (Fig 7).

## Discussion

To the best of our knowledge, this is the first study using a voxel-based DTI analysis to assess longitudinal alterations in white-matter tissue in both ESRD patients and healthy subjects over a 3-year period. Consistent with previous findings [8, 9, 17–19], the declines of the cognitive functions with associated alterations of the DTI indices were noted the ESRD patients. However, the longitudinal follow-up showed that the ESRD patients had no more interval changes of the DTI indices than the healthy controls. The findings suggest that the DTI indices in the ESRD patients undergoing regular hemodialysis were in a relatively stable condition, as the healthy controls.

In the cross-sectional analysis of our study, the higher diffusivity indices, including axial, radial and mean diffusivities, and the lower FA of white matter in the ESRD patients were noted than that in the healthy subjects in both the first and the follow-up scans. The findings of these microstructural alterations in ESRD patients were consistent with the previous studies about ESRD patients [8, 9, 17–19]. The decrease of FA may suggest the axonal degeneration or demyelination caused by ESRD and the decrease of the diffusivity indices may reflect the loss of axon and myelin [16, 20, 21]. However, in the follow-up scan, the extents of altered white-matters in the ESRD patients were slightly less than that in the first scan, especially in AD and FA values. Because FA in an indicator of white matter integrity, the results suggested that the 3-year hemodialysis did not worsen the white matter integrity in the ESRD patients.

In the interaction analysis (flexible factorial design), significant alterations of the DTI indices, including increased diffusivities and decreased FA, were noted in multiple white-matter regions only in the healthy controls after 3 years. The white-matter alterations in healthy controls were likely associated with normal aging as reported in the previous studies [22, 23]. However, when lowering statistical criteria to *P* < 0.05 and cluster > 100 voxels, slight and scattered white-matter alterations could be observed in the ESRD patients but more alterations with larger extent were also detected in white matter tissues of healthy controls. Hence, it is likely that ESRD patients with long-term hemodialysis may have similar degenerative alterations of white matter to healthy controls. As the ESRD patients should have had similar white-matter alterations to the age-matched control subjects due to normal aging over the period of 3 years, the unchanged or less altered the DTI indices in the ESRD patients indicated that an additional 3-year regular hemodialysis helped maintain white matter integrity during the period of this study.

In terms of dialysis duration, multiple regression analysis showed positive correlations with diffusivities and negative correlation with FA in white matter tissues of the ESRD patients, which were consistent with the previous DTI findings [8, 9]. In the cross-sectional analysis, the significant correlations between the DTI indices and the dialysis duration were mainly noted in the frontal lobes, which were also mentioned in the previous DTI findings [9, 17–19]. The finding suggests that the worsening of white matter integrity in the frontal lobes of the ESRD patients undergoing hemodialysis may be responsible for the decline of cognitive functions. However, the longitudinal analysis of our study revealed that the interval change of the DTI indices in frontal lobes of the ESRD patients was not significant. In our study, the dialysis duration (7.2 ± 3.4years) is longer than the follow-up interval (3 years) and the longer dialysis time may be the major variable causing the different results between the multiple regression analysis for dialysis duration and the longitudinal analysis for interval changes. The findings may emphasize that the 3-year regular hemodialysis did not cause significant white matter alterations and toxic effect of ESRD may be the major factor for the alteration of the DTI indices in the white matter tissues.

The patho-etiology of brain injury in ESRD is complex and controversial. The correlations between the serum urea levels and the alteration of the DTI indices in the ESRD patients in the previous studies [17, 18] may suggest that the cytotoxic effect of severe azotemia may be one of the major disease entities of brain damage in the ESRD patients. For the ESRD patients, hemodialysis can reduce the toxin level. However, some clinical complications may be noted in the initiation of hemodialysis and the interstitial edema of brain was identified as the significantly increased diffusivities of the brain tissue in the diffusion MR images after the hemodialysis immediately [7]. Nevertheless, the enrolled ESRD patients in our study have a long history of hemodialysis (mean duration of 10.2 years in the follow-up scan) and the MRI scan was performed two days after hemodialysis, so the effect of immediate hemodialysis, if any, could be neglected and the white-matter alterations observed in the ESRD patients were mainly due to ESRD itself.

Regarding cognitive function, the ESRD patients exhibited significantly lower CASI scores than healthy controls at both scans, suggesting that the patients had impaired cognitive functions which were likely associated with the white-matter alterations. Although the longitudinal analysis did not show a significant decrease of CASI scores in both groups, slightly more decline of CASI scores was noted in healthy controls than the ESRD patients after three years, in line with the results of DTI analysis that more significant white matter alterations were detected, especially in bilateral frontal lobes, in healthy controls than the ESRD patients after 3 years.

Furthermore, the significant interval white matter demyelination, characterized as increased RD but unchanged AD, was detected in bilateral anterior frontal lobes, left posterior corona radiata, and left forceps major of healthy controls. In these regions with significant demyelination, Pearson’s correlational analysis demonstrated that only RD value was significantly negatively correlated with the CASI scores. The findings suggest that RD is more closely associated with cognitive functions than other DTI indices. In addition, the white matter changes were significantly associated with age in multiple white matter regions in both the ESRD patients and the healthy subjects. Consistent with previous findings, the results demonstrated that white matter changes were age-dependent in both groups. However, more white matter regions with significant correlations were revealed in the ESRD patients than in the healthy subjects, suggesting that the ESRD patients exhibited more white matter changes due possibly to both normal aging and toxic effect of ESRD.

This study has some limitations. First, the study population was small because of the inclusion and exclusion criteria (young participants without pre-existing brain lesions and diabetes mellitus, and images scanned on the same scanner at two time points separated by 3 years). Lost to follow-up of some participants resulted in small sample size and some differences between the two groups (such as gender and age). Second, this study did not scan the ESRD patients before undergoing hemodialysis, so the influence of ESRD alone cannot be assessed in this study. Third, the neurological complications in ESRD are complicated and only cognitive function test was performed in our study. So, we could not provide the correlations between DTI and other neurological complications (e.g., depression, anxiety, dementia). Fourth, it has been known that the accuracy of voxel-wise analysis relies on the performance of the registration method in spatial normalization. This study performed both linear affine and non-linear demon registrations to reduce the global and local difference between the individual and template brain images in a voxel-based analysis, in order to minimize registration errors. Finally, DTI data were acquired with a 4-mm slice thickness, which led to more prominent partial volume effects in the through-plane direction and possibly affected the results.

## Conclusions

In the cross-sectional DTI analysis, white matter integrity was significantly lower in the ESRD patients than in the healthy controls; however, in the longitudinal voxel-based DTI analysis, less white-matter alterations were noted in the ESRD patients than that in healthy participants after a 3-years follow-up. In conclusion, poorer white matter integrity and cognitive function are noted in ESRD patients. The toxic effect of ESRD may be the major factor for white matter alterations in the ESRD patients, but 3-year regular hemodialysis did not cause significant white matter alterations.

## Supporting information

S1 Fig Multiple regression analysis for correlations between white matter alterations and age in healthy controls. Red-yellow colors show the regions with significant positive correlations between AD (a-c), RD (d-f), MD (g-i) and age, and a significant negative correlation between FA (j-l) and age. Color bars in the right-hand side indicate T-value.
(TIF)

S2 Fig Multiple regression analysis for correlations between white matter alterations and age in ESRD patients. Red-yellow colors show the regions with significant positive correlations between RD (a-c), MD (d-f) and age, and a significant negative correlation between FA (g-i) and age. Color bars in the right-hand side indicate T-value.
(TIF)

S1 Table Group comparisons of the DTI indices between patient and control groups at the first scan. The table shows MNI coordinates of regions with significantly different AD, RD, MD, and FA values of the first scan between the two groups.
(PDF)

S2 Table Group comparisons of the DTI indices between patient and control groups at the second scan 3 years later. The table shows MNI coordinates of regions with significantly different AD, RD, MD, and FA values of the second scan between the two groups.
(PDF)
