# Supplementary figures and images for: A preliminary report of longitudinal white matter alterations in patients with end-stage renal disease: A three-year diffusion tensor imaging study
Source: PLoS One. 2019 Apr 30;14(4):e0215942. doi: 10.1371/journal.pone.0215942 (PMC6490894; doi:10.1371/journal.pone.0215942)

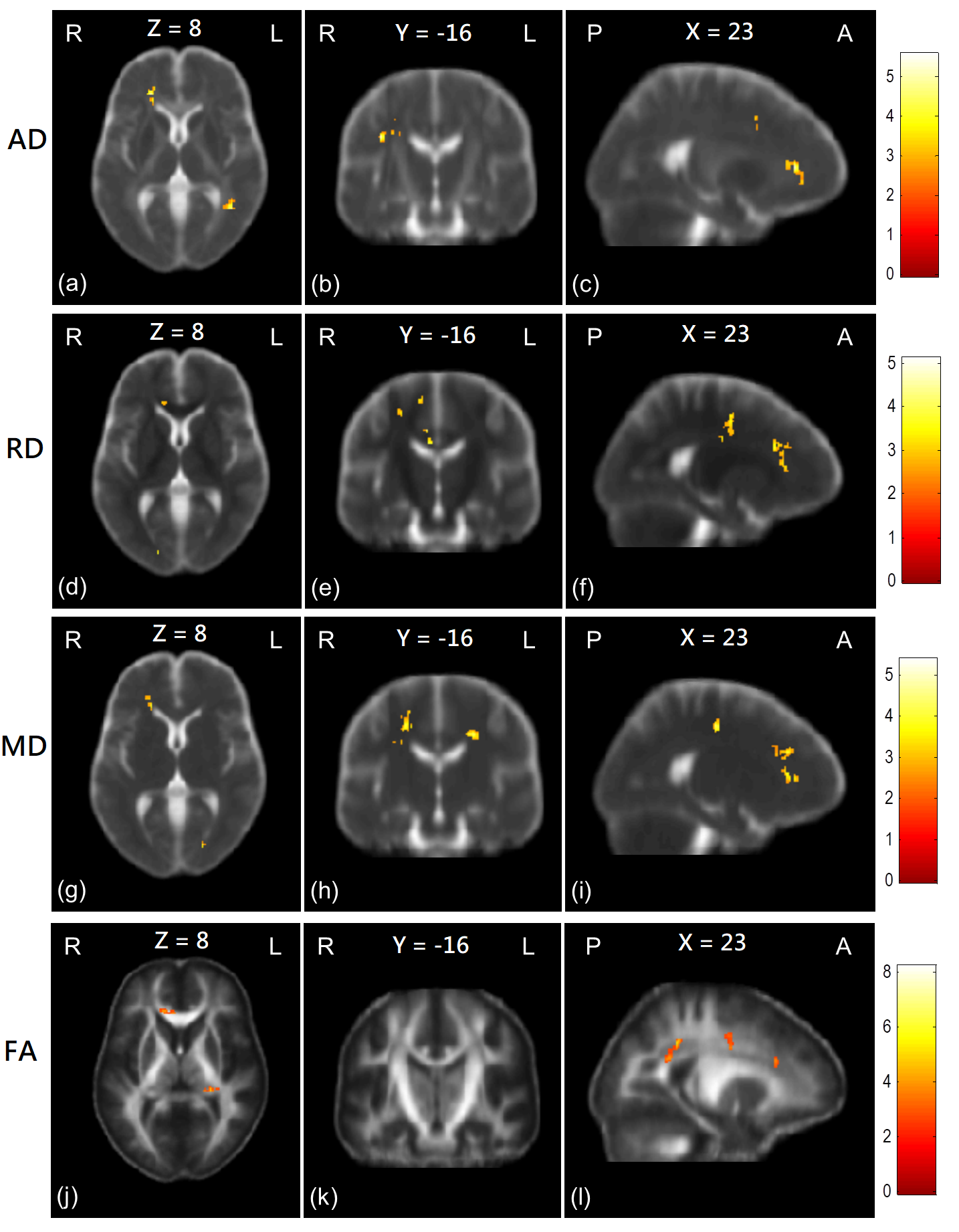

Supplement: S1 Fig — Red-yellow colors show the regions with significant positive correlations between AD (a-c), RD (d-f), MD (g-i) and age, and a significant negative correlation between FA (j-l) and age. Color bars in the right-hand side indicate T-value. (TIF) [file pone.0215942.s001.tif]

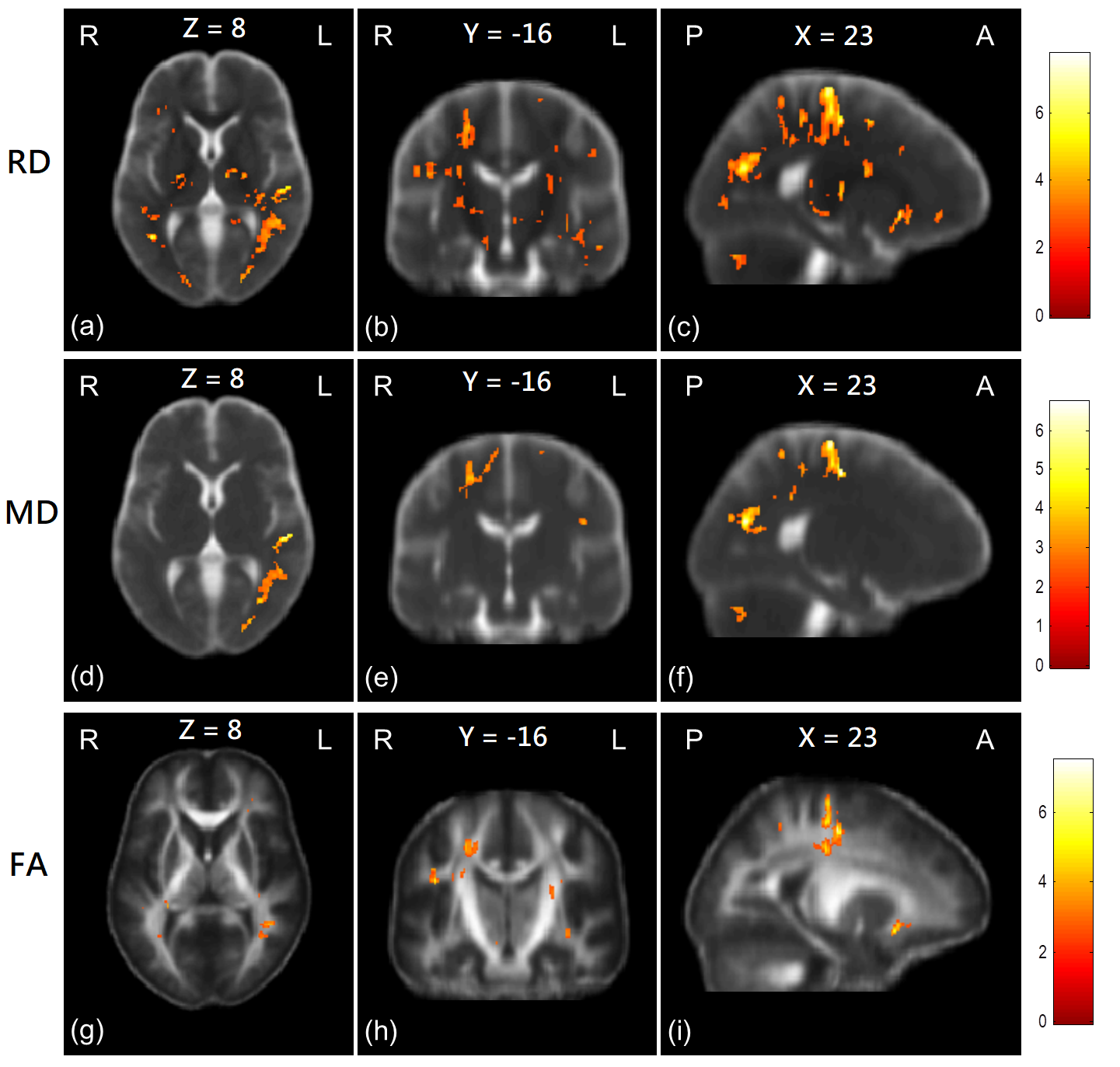

Supplement: S2 Fig — Red-yellow colors show the regions with significant positive correlations between RD (a-c), MD (d-f) and age, and a significant negative correlation between FA (g-i) and age. Color bars in the right-hand side indicate T-value. (TIF) [file pone.0215942.s002.tif]
