# Supplementary material for: A preliminary report of longitudinal white matter alterations in patients with end-stage renal disease: A three-year diffusion tensor imaging study
Source: PLoS One. 2019 Apr 30;14(4):e0215942. doi: 10.1371/journal.pone.0215942 (PMC6490894; doi:10.1371/journal.pone.0215942)
Supplement: S1 Table — The table shows MNI coordinates of regions with significantly different AD, RD, MD, and FA values of the first scan between the two groups. (PDF) [file pone.0215942.s003.pdf]

Table S1. MNI coordinates of regions with significantly different AD, RD, MD, and FA values of the first scan between ESRD patients and healthy controls.

	MNI Coordinate (mm)			AD * 10^{-3} mm^2/s	
Brain Regions	X	Y	Z	ESRD	Normal
Lt. ACR	-31	25	26	1.093 ± 0.035	0.976 ± 0.021
				RD * 10^{-3} mm^2/s	
Lt. ACR	-14	35	36	0.694 ± 0.072	0.576 ± 0.051
Rt. PCR	35	-61	18	0.743 ± 0.064	0.602 ± 0.066
				MD * 10^{-3} mm^2/s	
Rt. PCR	35	-61	18	0.842 ± 0.037	0.754 ± 0.026
Lt. ACR	-29	35	8	0.820 ± 0.035	0.731 ± 0.020
GCC	6	20	18	0.939 ± 0.053	0.824 ± 0.030
				FA	
Rt. Fminor	12	32	-9	0.343 ± 0.028	0.422 ± 0.034
Rt. PCR	33	-52	27	0.360 ± 0.022	0.445 ± 0.023
Lt. ACR	-31	25	13	0.303 ± 0.029	0.378 ± 0.022
Lt. SS	-41	-28	-14	0.395 ± 0.031	0.464 ± 0.021

ACR=anterior corona radiata; Fminor=forceps minor; GCC=genu of corpus callosum; PCR=posterior corona radiata; SS=sagittal stratum

Lt = left; Rt = right
