# Supplementary material for: A preliminary report of longitudinal white matter alterations in patients with end-stage renal disease: A three-year diffusion tensor imaging study
Source: PLoS One. 2019 Apr 30;14(4):e0215942. doi: 10.1371/journal.pone.0215942 (PMC6490894; doi:10.1371/journal.pone.0215942)
Supplement: S2 Table — The table shows MNI coordinates of regions with significantly different AD, RD, MD, and FA values of the second scan between the two groups. (PDF) [file pone.0215942.s004.pdf]

Table S2. MNI coordinates of regions with significantly different AD, RD, MD, and FA values of the second scan between ESRD patients and healthy controls.

Brain Regions	MNI Coordinate (mm)			RD * 10^{-3} mm^2/s	
	X	Y	Z	ESRD	Normal
Lt. PCR	-51	-40	28	0.833 ± 0.171	0.645 ± 0.078
				MD * 10^{-3} mm^2/s	
Rt. SCR	28	-2	37	0.863 ± 0.099	0.742 ± 0.026
Lt. PCR	-42	-52	44	1.033 ± 0.046	0.771 ± 0.062
Lt. SCR	-27	1	40	0.876 ± 0.073	0.757 ± 0.028
				FA	
Rt. ACR	30	15	20	0.327 ± 0.023	0.405 ± 0.024
Rt. Fmajor	23	-78	8	0.352 ± 0.035	0.447 ± 0.043
Rt. SS	37	-49	6	0.403 ± 0.037	0.487 ± 0.028
Lt. SCR	-43	-25	29	0.283 ± 0.029	0.363 ± 0.030

ACR=anterior corona radiata; Fmajor=forceps major; PCR=posterior corona radiata;

SCR=superior corona radiata; SS=sagittal stratum

Lt = left; Rt = right
